# The Future of Medical Benefit

**Published:** 1917-07-21

**Authors:** 


### NATIONAL INSURANCE ACT DEVELOPMENTS.

### The Future of Medical Benefit.

The change of attitude of the medical profession, taken as a whole, towards national health insurance, which has been brought about through practical experience of the scheme, is one of the most remarkable features in the development of the administration of the Act. Our reason for referring to the matter now is that a most excellent report has just been issued by the British Medical Association, and published in the *British Medical Journal*, in which the administration of medical benefit under the Act is examined, and in which various suggestions are put forward for the improvement of the system, and for securing to the population as a whole, and not only those covered by insurance, an adequate Medical Service.

The report is, at the moment, of an interim character; that is to say, it is to be considered by local branches of the Association, and it will not be until after the results of the deliberations of the local branches have been summarised that the final report will be submitted to the Council and representative body of the Association, to become the accepted policy of the medical profession with regard to future changes that may be proposed in the system of national health insurance. The work has, however, been done so well that there is no reason to suppose that the final report will differ in any material degree from the interim report now submitted. Moreover, seeing that the report, as it now stands, is the result of inquiries made by the National Insurance Acts Committee of the British Medical Association from each branch and division of the Association, and each local medical and panel committee, it may be said that it already represents to a large extent the considered opinion of the local branches of the Association, and of those most intimately connected with the administration of medical benefit.

The Committee is to be congratulated on the expedition with which the work has been completed, for we learn from the report that it was not until January 17 last that it decided to ask the local branches and panel committees to appoint thoroughly representative sub-committees to consider the present system of national health insurance so far as it affects the relation of the medical profession to the public health and treatment of disease, and to make suggestions for its improvement. In order that the investigation might be as complete and thorough as possible inquiries were made on certain specific points, and the opinion of doctors on military service was also sought as far as this was found to be practicable.

General Opinion of the Medical Profession.

The Committee states that "under the present war conditions the response to its inquiries have been surprisingly good." The report then proceeds: "The degree of unanimity so far disclosed is somewhat remarkable. On a subject which five years ago was the most highly controversial that had ever been before the profession, and which still in some places, and everywhere in some of its aspects, excites argument, it is found (1) that many matters which at the beginning of the controversy gave rise to most apprehension have assumed a position of quite minor importance; (2) that the general system by which the State provides medical advice and treatment under the insurance scheme is in the main approved, and that criticisms have a tendency to concentrate on a comparatively few points, which, though of great importance and indeed vital to smooth working, are, after all, matters of detail which ought to be capable of adjustment; and (3) that there is a large body of opinion in favour of the extension of the health insurance system both to kinds of treatment not at present provided for, and to classes of persons at present excluded therefrom."

The Committee continues: "The replies received show that the profession entirely agrees that medical benefit is inadequate in extent, and that the idea is still prevalent in some quarters that the profession is being asked to give more than the State is paying for." On this aspect of the matter the Committee make the following trenchant observation: "The system will never be satisfactory either to the public or to the medical profession until both are convinced that the conditions of service and of payment are such that the practitioner has no reason for making any difference between the patient who pays private fees for attendance and the patient for whose attendance the State is responsible."

Here we have the ultimate aim of national insurance expressed in its most concrete form. National insurance is not charity, and the stigma of poor relief must not be allowed to attach itself thereto in any shape or form.

Under national insurance the insured person benefits in the provision of facilities to enable him or her to enjoy good health; the employer benefits by the good health of his workpeople; and the community as a whole reaps many advantages, both social and economic, as a direct result of the good health of the workers. Each class, therefore, has to pay its quota towards the provision of the facilities for the treatment of sickness and disease, but it would not be fair to expect the medical profession to undertake the duties of treatment and attendance without adequate remuneration for the services rendered.

### The Opinion of Insured Persons.

The Committee desired to obtain some idea of the general opinion of insured persons as to the administration of medical benefit, and as far as this can be gauged from the opinions expressed to the doctors, the principal complaints are with regard to delay in obtaining medical cards and to certification of incapacity. Complaint is also made as to the inadequacy of the surgery and waiting-room accommodation which some insurance practitioners possess. Owing to this inadequacy of accommodation, discredit has been thrown on the whole system in certain areas, as it has been found impossible to avoid invidious comparisons with decent private practice. In spite of these complaints, the Committee is of opinion that the insured persons are generally satisfied with the system and service, for whole classes of them are receiving treatment to an extent they had never previously thought of, and it is gratifying to learn that "in thousands of cases relations of confidence are being established between practitioner and insured patient."

### The Improvement of the System.

Interesting as the survey of the existing system undoubtedly is, it will be admitted that the suggestions put forward for the improvement of the system are infinitely the more important. In this connection the Committee has considered the question from the point of view of an improvement of the existing panel system, as against the institution of a new system on the lines of a State medical service consisting of salaried medical officers. The result of this consideration is that, though there must be some extension of the number of salaried medical officers in connection with preventive medicine, the treatment of individuals, except in institutions, is best carried out on a system under which the practitioners are paid by some method dependent either upon the actual items of work done, or upon the number of persons for whom they accept responsibility. The Committee, therefore, rejects the idea of a salaried medical service and puts forward suggestions with a view to removing some at least of the acknowledged imperfections of the present panel system. The most important of these suggestions is that the scope of medical benefit should be extended so that it should no longer be confined to such treatment as can be given by the ordinary general practitioner. It is anticipated that if the benefit were extended to include specialist services, thus enlisting the support and co-operation of a number of eminent medical men not engaged in general practice, a large number of general practitioners, who have so far held aloof from all connection with the system, would be attracted to it. The Committee also suggests that there is considerable scope for improvement in the constitution of Insurance Committees, it being desirable that the medical profession should be given larger representation thereon, while at the same time there should be a reduction in the present preponderance of approved society representatives.

### Extension of Classes of Persons Entitled to Benefit.

After referring to certain administrative defects in the present scheme, in connection with deposit contributors and persons whose incomes exceed the limit of £160—which defects are in the nature of matters of detail—the Committee go on to suggest that the scheme might with advantage be extended so as to abolish the "parish doctor" as such. In other words, the unification of treatment under one authority is advocated. This may and should be possible upon the institution of a Ministry of Health. Another class of persons to whom it is suggested that the advantages of medical benefit should be extended are the dependants of insured persons. It is not proposed, however, that this should be done indiscriminately, but rather to allow only those to participate who really need public aid for this purpose. Certain practical and administrative difficulties would at once arise in carrying out such a proposal, but it should be possible for these to be overcome in view of the fact that such dependants—mainly women and children—are as much in need of improved medical attention as are those on whom they are dependent.

### Proposed Extension of Medical Benefit.

Not only does the Committee propose the extension of the classes of persons to whom medical benefit is to apply; it also suggests the extension of the benefit itself so as to include as a right, and under proper safeguards, the provision of (1) a consultant and specialist service, (2) institutional treatment, (3) pathological and clinical laboratory facilities, (4) *x*-ray provision, both for diagnosis and treatment, (5) special forms of treatment, such as massage and electricity, (6) dental treatment, (7) a nursing service, and (8) advice with regard to pregnancy and attendance at confinement by a midwife, with emergency attendance by a practitioner.

It is suggested that all these services should be outside the contract for ordinary domiciliary treatment and that special arrangements should be made for the remuneration of practitioners rendering them. The Committee would not, however, exclude the ordinary general practitioner from rendering such services when qualified to do so.

The treatment in hospitals and special clinics, not at present provided under the terms of medical benefit, is recognised as of vital importance, and the proposal made by the Committee for linking up the work at such institutions with domiciliary attendance is to be welcomed, and it is to be hoped that the means will be found for carrying it into effect.

